# Factors associated with regularity and length of menstrual cycle: Korea Nurses’ Health Study

**DOI:** 10.1186/s12905-022-01947-z

**Published:** 2022-09-01

**Authors:** Sihan Song, Hansol Choi, Yanghee Pang, Oksoo Kim, Hyun-Young Park

**Affiliations:** 1 Division of Population Health Research, Department of Precision Medicine, Korea National Institute of Health, Cheongju, Republic of Korea; 2 Department of Nursing, Baekseok Culture University, Cheonan, Republic of Korea; 3 College of Nursing, Ewha Womans University, Seoul, Republic of Korea; 4 Department of Precision Medicine, Korea National Institute of Health, Cheongju, 28159 Republic of Korea

**Keywords:** Menstrual cycle, Nurses, Reproductive history, Lifestyle, Environmental factor

## Abstract

**Background:**

Menstrual cycle characteristics are linked to reproductive function and long-term health outcomes. This study aimed to evaluate menstrual cycle patterns, characterized by regularity and length, and associated factors among women in the Korea Nurses’ Health Study.

**Methods:**

A total of 9335 premenopausal women aged 22–45 years were included in this cross-sectional study. Regularity and length of menstrual cycles were self-reported, and their associations with reproductive, lifestyle, and occupational factors were examined using binomial and multinomial logistic regression models. Adjusted least-square means of menstrual distress, depressive symptoms, stress, fatigue, anxiety, and sleep problems were estimated according to menstrual cycle characteristics using generalized linear models.

**Results:**

Twenty-one percent of nurses reported having irregular menstrual cycles (variability > 7 days). Ten percent, 64%, and 26% had menstrual cycle length of < 26, 26–31, and 32–50 days, respectively. Variability and length of cycles decreased with age and increased with age at menarche. Parous women showed low tendency of irregular cycles. Women with body mass index (BMI) > 25 kg/m^2^ had higher odds of irregular (odds ratio [OR] 1.68; 95% confidence interval [CI] 1.40–2.03) and long cycles (OR 1.31; 95% CI 1.08–1.58) than those with BMI 18.5– < 23 kg/m^2^. Irregular cycles were less common in women performing vigorous physical activity, but more common in those with prolonged standing or frequent heavy lifting at work. Frequent rotating night shift was associated with irregular cycles among nulliparous women. Levels of menstrual and premenstrual distress, depressive symptoms, perceived stress, physical and mental fatigue, anxiety, and sleep problems were higher in women with irregular cycles than in those with regular cycles (*p* < 0.001, each).

**Conclusions:**

The study suggests that irregular and long menstrual cycles are associated with reproductive, lifestyle, and occupational factors; also with menstrual distress and perceived health status. Our findings contribute to a better understanding of potential risk factors for menstrual dysfunction, and thus, may help improve women’s health.

**Supplementary Information:**

The online version contains supplementary material available at 10.1186/s12905-022-01947-z.

## Background

The menstrual cycle is regulated by a complex interaction of hormones produced by the hypothalamus, pituitary, and ovaries [1]. The menstrual cycle is divided into follicular and luteal phases, separated by ovulation, and regularity and length of cycles are considered as markers of endocrine function and reproductive health [2, 3]. Furthermore, accumulating evidence suggests that menstrual cycle characteristics are associated with long-term health outcomes, probably through mechanisms involving hormonal imbalances and metabolic disturbances. Long and/or irregular cycles have been associated with increased risk of type 2 diabetes [4, 5], coronary heart diseases [6, 7], ovarian cancer [8], and premature mortality [9].

Menstrual disorders include disruption of menstrual patterns, ovarian dysfunction and menstrual pain, and are common in women of reproductive age [3]. In previous studies, the prevalence of menstrual irregularities in adult women generally ranged from 5 to 30% [10–16]. Menstrual cycle characteristics have been associated with age, endocrine conditions, reproductive factors (e.g., age at menarche and parity), and modifiable lifestyle factors, including weight, physical activity, and stress [2, 3]. Menstrual function may be associated with working conditions, especially shift work, and occupational chemical exposures [3]. A recent meta-analysis of observational studies reported that women working rotating shifts were more likely to experience menstrual irregularity than those working fixed day shifts [17]. There is limited evidence on menstrual cycle characteristics among Korean women who work rotating shifts. In a previous Korean study, insomnia was associated with increased odds of irregular menstrual cycles among newly-employed shift-working nurses [18]. In the Korea Nurses’ Health Study (KNHS), an ongoing prospective study of Korean female nurses, polycystic ovary syndrome (PCOS) was more prevalent among nurses with shift work [19]. In that study, obesity was significantly associated with irregular menstrual cycles among women with PCOS.

Identifying menstrual patterns and associated factors is the first step to understand women’s health and examine the long-term effects of reproductive experience. Moreover, more data on menstrual cycle characteristics from different populations should be obtained to explore factors contributing to variations in menstrual patterns. Nursing is a physically and emotionally demanding profession; therefore, investigating factors affecting menstrual cycle characteristics of nurses may provide valuable insights regarding reproductive health of working women.

This study aimed to examine the association of reproductive, lifestyle, and occupational factors with menstrual cycle regularity and length in nurses enrolled to the KNHS. Furthermore, we investigated whether the levels of menstrual distress and perceived health statuses differed according to menstrual cycle characteristics.

## Methods

### Study population

The KNHS is a web-based cohort study of Korean female nurses aged 20–45 years at enrollment, initiated in 2013 [20]. The KNHS was designed to investigate the effects of occupational, environmental, and lifestyle factors on women’s health. A total of 20,613 women responded to the baseline questionnaire (module 1) between July 2013 and November 2014. Participants were invited through a text message to continue with subsequent online surveys. Six survey modules (modules 2–7) were subsequently opened to participants between 2014 and 2019. From the module 8 started in 2019, participants continue to be followed up via annual questionnaires; and module 10 was administered beginning October 2021. Updated information was obtained by questionnaires, including data on disease history, job status, and reproductive factors. Women who were pregnant or who recently given birth were invited to respond to additional pregnancy modules. Detailed description of the study design and protocol are given elsewhere [20].

In the module 3, questions on menstrual cycle characteristics were included and 12,851 women responded to the survey. The median time interval between modules 1 and 3 was two years. For the current analysis, we excluded women aged > 45 years to reduce variation from perimenopausal status (*n* = 275) or those who had reached menopause (*n* = 75) at the time of answering the module 3. We also excluded women who were pregnant (*n* = 497), had given birth or breastfed within the last 6 months (*n* = 310), had undergone hysterectomy (*n* = 126) or oophorectomy (*n* = 180), who currently used hormonal contraceptives (*n* = 438), reported no periods (*n* = 156), were diagnosed with cancer (*n* = 248), or had missing data on menstrual cycle characteristics (*n* = 17). Of 10,882 eligible participants, we further excluded women who reported endometriosis (*n* = 270), uterine fibroids (*n* = 727), or PCOS (*n* = 702) to reduce confounding of gynecological conditions. Finally, 9335 women were included in this cross-sectional analysis. When we examined association of potential risk factors with menstrual cycle length, we excluded women who reported cycles longer than 50 days or too irregular to estimate (*n* = 292); 97% of them had usually or always irregular menstrual cycles.

### Data collection

The menstrual cycle patterns were characterized by cycle regularity and length. The menstrual cycle characteristics were assessed based on the questionnaire used in Nurses’ Health Study 3 (NHS3), an ongoing prospective cohort study of the US and Canadian nurses [15, 20]. In the module 3, participants were asked to report the current menstrual cycle regularity as very regular (within 3 days), regular (within 5–7 days), usually irregular, or always irregular. Participants also reported their usual menstrual cycle length as < 21, 21–25, 26–31, 32–39, 40–50 days, or > 50 days or too irregular to estimate. Additionally, data on age at menarche, parity, menstrual distress, physical activity, and medical disease history were collected in the module 3. Menstrual distress was assessed by the Menstrual Distress Questionnaire Form-C (MDQ-C) [21, 22]. The MDQ-C is a list of 46 symptoms that are rated on a five-point scale ranging from “no experience of symptoms” to “severe” and are clustered into eight symptom domains as follow: pain, water retention, autonomic reactions, negative affect, impaired concentration, behavior change, arousal, and control. A higher score indicates more severe symptoms, except in case of arousal. Symptoms are separately assessed for the most recent cycle during the menstrual, premenstrual (four days before menstrual flow), and intermenstrual phases. In the present study, symptoms during the menstrual and premenstrual phases were measured, and scores for each phase were calculated using the seven negative-symptom domains (41 items). The scale has a reliability coefficient (Cronbach’s alpha) of 0.91 for the menstrual phase and 0.92 for the premenstrual phase. The average amount of time spent per week for physical activities over the past year was obtained from participants using a structured questionnaire [23], and a metabolic equivalent (MET) value was assigned to each physical activity based on the classification by Ainsworth et al. [24]. A sum of weighted values for recreational physical activities requiring ≥ 6 METs (e.g., running, bicycling, and swimming) was used to estimate MET-hours/week for vigorous activity [23].

In the module 1–the initial baseline survey–participants were asked about demographic information, disease history, reproductive factors, current weight and height, work conditions, perceived health status, and lifestyle factors including smoking status. Body mass index (BMI) was calculated as a ratio of body weight (kg) to height squared (m^2^), and categorized using the World Health Organization Asia–Pacific criteria as: underweight (< 18.5 kg/m^2^), normal weight (18.5– < 23 kg/m^2^), overweight (23– < 25 kg/m^2^) and obese (≥ 25 kg/m^2^) [25]. For work conditions, current work schedules were collected as an average frequency of rotating night shifts per month over the past year. Physical demands at work on average over the past month were collected as time spent on feet (standing or walking; hours/day) and frequency of heavy lifting (physical load of 10 kg; times/day). The levels of perceived stress, depressive symptoms, physical and mental fatigue, anxiety, and sleep problems were measured using the four-item Perceived Stress Scale (PSS-4) [26], Patient Health Questionnaire-9 [27], Chalder Fatigue Scale [28], the six item Spielberger State-Trait Anxiety Inventory [29], and Jenkins Sleep Questionnaire [30], respectively. The scores for each instrument were calculated according to protocol for these methods, with higher scores representing greater symptoms. The Cronbach’s alpha values of those instruments in our study were ≥ 0.70, except for the PSS-4 (0.53). Additionally, participants were asked to scale their health as excellent, good, fair, poor, or very poor.

In the module 2–the first follow-up survey–the frequency and amount of the three types of alcoholic beverages over the past year were collected in the food frequency questionnaire (FFQ). Total alcohol consumption (g/day) was calculated as the sum of frequency of drinking multiplied by the alcohol content (ethanol) of the individual alcoholic beverages. Coffee consumption (cups/day) on average over the past year was also calculated using the FFQ.

### Statistical analysis

Descriptive analyses of the characteristics of participants were performed according to the regularity and length of menstrual cycles. Menstrual cycle regularity was categorized as “regular (cycle variability within 7 days)” and “irregular” and menstrual cycle length was categorized as “short (< 26 days),” “moderate (26–31 days),” and “long (32–50 days)” [1, 15]. Logistic regression was performed to estimate odds ratios (ORs) and 95% confidence intervals (CIs) for the association of reproductive, lifestyle factors, and working conditions with menstrual cycle characteristics. Binomial logistic regression was used to estimate the odds of irregular cycles and multinomial logistic regression was used to calculate the odds of short and long cycle length compared with moderate cycle length, in relation to potential determinants. Generalized linear models were used to estimate least-square means (LSmeans) and standard errors (SEs) of menstrual distress and perceived health status according to the menstrual cycle characteristics. Covariates were selected based on prior studies [2, 3, 10, 11, 13, 15, 31–37] and the change-in-estimate method [38]. Multivariable models were adjusted for age (years), age at menarche (≤ 12, 13, 14, or ≥ 15 years), parity (nulliparous, 1, or 2+), BMI (kg/m^2^), vigorous physical activity (none or tertiles), and alcohol consumption (0, < 5, 5– < 15, or ≥ 15 g/day). P for trend was calculated by assigning the midpoint of each category and using as a continuous variable in the model. Sensitivity analyses were performed by excluding women aged > 40 years or restricting analyses to nulliparous women. We also confirmed associations among participants with a time interval of < 2 years between module 1 and 3.

All statistical analyses were performed using SAS statistical software version 9.4 (SAS Institute Inc., Cary, NC). A two-sided *p* < 0.05 was considered to be statistically significant. Results of Bonferroni correction were additionally reported for multiple comparisons.

## Results

The characteristics of 9335 premenopausal women included in the study are presented in Table 1. The mean age of participants was 30.83 (range 22–45) years. A majority of the women were nulliparous (70.97%), never smokers (97.21%), and worked rotating night shifts (70.68%). A total of 1976 (21.17%) women reported irregular menstrual cycles; and 507 (5.43%) reported that their cycles were always irregular. Of women with regular cycles, 71.45% had a cycle length of 26–31 days. Of the 9043 participants who reported menstrual cycle length of ≤ 50 days, 886 (9.80%), 5794 (64.07%), and 2363 (26.13%) reported menstrual cycle lengths of < 26 days, 26–31 days, and 32–50 days, respectively. Ninety-one percent of the women with menstrual cycle length of 26–31 days had a regular cycle. Women with irregular cycles were more likely to be younger, have late age at menarche, be nulliparous, or drink alcohol, and less likely to drink coffee than women with regular cycles. Moreover, women with irregular cycles were more likely to work rotating night shifts, perform long hours of standing work, or require frequent heavy lifting at work than those who reported regular cycles. Similar results were observed among women who reported long cycle lengths compared with women who reported moderate cycle length.

**Table 1:** Characteristics of participants in the Korea Nurses’ Health Study according to menstrual cycle regularity and length

Characteristics^a^	All (n = 9335)	Menstrual cycle regularity		*p* value^b^	Menstrual cycle length^c^			*p* value^b^
		Regular	Irregular		< 26 days	26–31 days	32–50 days	
Number		7359 (78.83)	1976 (21.17)		886 (9.80)	5794 (64.07)	2363 (26.13)	
Age, years	30.83 ± 5.64	31.24 ± 5.71	29.31 ± 5.12	< 0.001	31.39 ± 6.34	31.33 ± 5.79	29.61 ± 4.76	< 0.001
*Menarche age, years*								
≤ 12	2139 (22.91)	1709 (23.22)	430 (21.76)	< 0.001	205 (23.14)	1326 (22.88)	532 (22.52)	0.435
13	2544 (27.25)	2033 (27.63)	511 (25.86)		249 (28.10)	1599 (27.60)	631 (26.70)	
14	2157 (23.11)	1731 (23.52)	426 (21.56)		181 (20.43)	1357 (23.42)	556 (23.53)	
≥ 15	2495 (26.73)	1886 (25.63)	609 (30.82)		251 (28.33)	1512 (26.10)	644 (27.25)	
*Parity*								
Nulliparous	6620 (70.97)	5019 (68.25)	1601 (81.10)	< 0.001	617 (69.72)	3942 (68.08)	1816 (76.92)	< 0.001
1	1023 (10.97)	860 (11.69)	163 (8.26)		100 (11.30)	675 (11.66)	222 (9.40)	
2+	1685 (18.06)	1475 (20.06)	210 (10.64)		168 (18.98)	1173 (20.26)	323 (13.68)	
Body mass index, kg/m^2^	20.86 ± 2.68	20.86 ± 2.64	20.85 ± 2.82	0.599	20.62 ± 2.47	20.88 ± 2.62	20.80 ± 2.78	0.011
Vigorous physical activity, METs-hours/week	7.59 ± 21.16	7.59 ± 21.57	7.58 ± 19.56	0.611	7.25 ± 17.83	7.57 ± 22.00	7.65 ± 20.62	0.775
*Cigarette smoking*								
Never	9073 (97.21)	7160 (97.31)	1913 (96.86)	0.282	857 (96.73)	5642 (97.39)	2292 (97.04)	0.419
Ever	260 (2.79)	198 (2.69)	62 (3.14)		29 (3.27)	151 (2.61)	70 (2.96)	
*Alcohol drinking, g/day*								
None	3087 (34.65)	2450 (34.86)	637 (33.86)	0.017	271 (31.84)	1930 (34.92)	798 (35.34)	0.240
< 5	3448 (38.70)	2757 (39.23)	691 (36.74)		339 (39.84)	2172 (39.30)	849 (37.60)	
5– < 15	1295 (14.54)	1001 (14.24)	294 (15.63)		124 (14.57)	779 (14.09)	345 (15.28)	
≥ 15	1079 (12.11)	820 (11.67)	259 (13.77)		117 (13.75)	646 (11.69)	266 (11.78)	
Coffee consumption, cups/day	0.94 ± 1.24	0.94 ± 1.23	0.91 ± 1.27	0.007	0.97 ± 1.29	0.98 ± 1.26	0.84 ± 1.18	< 0.001
*Rotating night shift, nights/month*								
None	2734 (29.32)	2259 (30.73)	475 (24.09)	< 0.001	288 (32.58)	1788 (30.90)	597 (25.29)	< 0.001
< 7	3409 (36.57)	2673 (36.36)	736 (37.32)		309 (34.95)	2087 (36.06)	910 (38.54)	
≥ 7	3180 (34.11)	2419 (32.91)	761 (38.59)		287 (32.47)	1912 (33.04)	854 (36.17)	
*On feet at work, hours/day*								
≤ 4	2989 (32.02)	2442 (33.18)	547 (27.68)	< 0.001	281 (31.72)	1905 (32.88)	719 (30.43)	0.004
5–8	4254 (45.57)	3368 (45.77)	886 (44.84)		381 (43.00)	2671 (46.10)	1080 (45.70)	
≥ 9	2092 (22.41)	1549 (21.05)	543 (27.48)		224 (25.28)	1218 (21.02)	564 (23.87)	
*Heavy lifting at work, times/day*								
0	1590 (17.03)	1303 (17.70)	287 (14.53)	< 0.001	153 (17.27)	1034 (17.85)	356 (15.07)	0.038
1–5	5472 (58.62)	4329 (58.83)	1143 (57.84)		509 (57.45)	3390 (58.51)	1426 (60.35)	
≥ 6	2273 (24.35)	1727 (23.47)	546 (27.63)		224 (25.28)	1370 (23.64)	581 (24.58)	

*Missing data*: parity (*n* = 7), body mass index (*n* = 48), coffee consumption (*n* = 427), alcohol consumption (*n* = 426), cigarette smoking (n = 2), and rotating night shift (*n* = 12)

METs, metabolic equivalents

^a^Data expressed as mean ± standard deviation or number (percentage)

^b^Compared using Kruskal–Wallis, ANOVA, Mann–Whitney U-test, or t-test for continuous variables and Chi-square tests for categorical variables

^c^Excluding women who reported that their cycles are 51 + days or too long to estimate (n = 292)

**Fig. 1:**
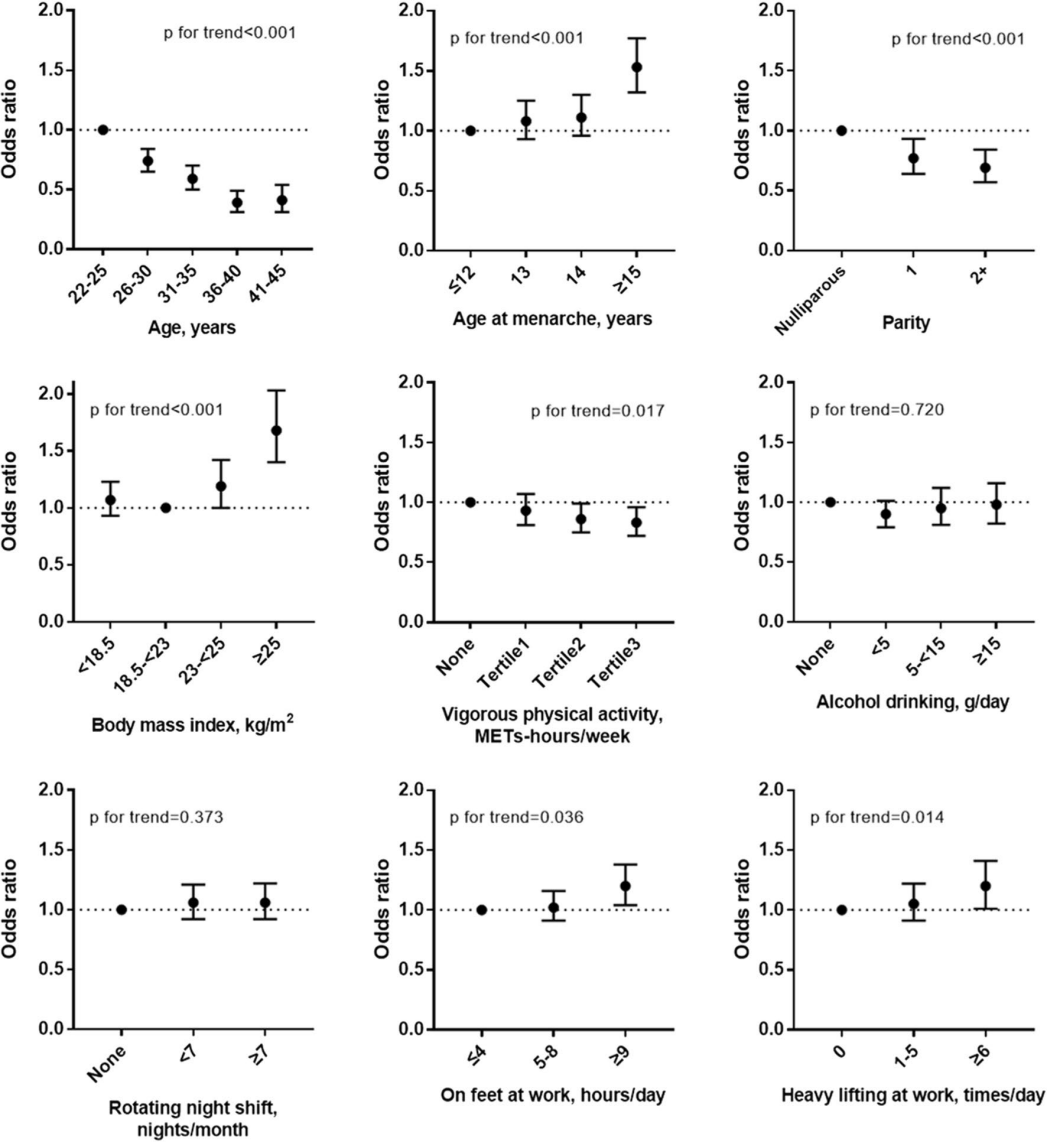
Associations of reproductive, lifestyle, and occupational factors with menstrual cycle irregularity in 9335 participants of the Korean Nurses' Health study. METs, metabolic equivalents. Compared to regular cycles defined as ≤ 7-day variability between cycles. Adjusted for age (years), age at menarche (≤ 12, 13, 14, or ≥ 15 years), parity (nulliparous, 1, or 2+), body mass index (kg/m^2^), vigorous physical activity (none or tertiles), and alcohol consumption (0, < 5, 5– < 15, or ≥ 15 g/day). Lines above and below the point estimates are 95% confidence intervals. The reference group is represented by simple black circle without confidence intervals

Adjusted associations of age, reproductive and lifestyle factors, and working conditions with irregular menstrual cycles are presented in Fig. 1. The odds of having irregular cycles decreased with increasing age (p for trend < 0.001). Late age at menarche was associated with higher odds of irregular cycles (≥ 15 vs. ≤ 12 years: OR 1.53; 95% CI 1.32–1.77), and higher parity was associated with lower odds of irregular cycles (2 + vs. nulliparous; OR 0.69; 95% CI 0.57–0.84). An approximate “J-shaped” association was observed between BMI and irregular menstrual cycles in the age-adjusted model (Additional file 1: Table S1). In multivariable-adjusted models, women with BMI ≥ 25 kg/m^2^ had significantly higher odds of irregular cycles than women with BMI 18.5– < 23 kg/m^2^ (OR 1.68; 95% CI 1.40–2.03). In terms of physical activity, women engaging in vigorous physical activity were less likely to have irregular cycles (Tertile3 vs. none; OR 0.83; 95% CI 0.72–0.96, p for trend = 0.017). However, the probability of having irregular cycles increased with increasing hours of standing work (≥ 9 vs. ≤ 4 h/day: OR 1.20; 95% CI 1.04–1.38) and frequent heavy lifting at work (≥ 6 vs. none: OR 1.20; 95% CI 1.01–1.41); p for trend < 0.05 for each. There were no significant associations of smoking status, alcohol and coffee consumption, and rotating night shifts with menstrual cycle regularity (Additional file 1: Table S1).

The multivariable-adjusted associations between potential risk factors and short or long menstrual cycle lengths are presented in Table 2. A strong inverse association was observed between age and long cycle lengths (p for trend < 0.001). Late age at menarche was significantly associated with higher odds of long cycle lengths (p for trend = 0.004). The odds of having long cycle lengths were higher in women with BMI ≥ 25 kg/m^2^ than in those with 18.5– < 23 kg/m^2^ (OR 1.31; 95% CI 1.08–1.58), and lower in women with higher levels of vigorous physical activity (Tertile3 vs. none: OR 0.83; 95% CI 0.73–0.96, *p* for trend = 0.034). Higher alcohol consumption was associated with higher odds of having short cycles and lower odds of having long cycles. The odds of having long cycle lengths decreased with increasing coffee consumption (p for trend = 0.030). For working conditions, prolonged standing was significantly associated with short cycle lengths (≥ 9 vs. ≤ 4 h/day: OR 1.27; 95% CI 1.04–1.56). There were no significant associations of parity, smoking status, rotating night shifts, and heavy lifting at work with menstrual cycle lengths.

**Table 2:** Associations of reproductive, lifestyle, and occupational factors with menstrual cycle length in 9043 participants of the Korean Nurses' Health study

Multivariable-adjusted ORs (95% CIs)^a^	No. of short/short or moderate	Short cycle length (< 26 days)	No. of long/moderate or long	Long cycle length (32–50 days)
*Age, years*				
22–25	180/1116	Reference	479/1415	Reference
26–30	288/2413	0.72(0.59–0.88)	1065/3190	0.97(0.85–1.11)
31–35	184/1473	0.82(0.64–1.04)	495/1784	0.73(0.62–0.86)
36–40	123/1025	0.85(0.63–1.13)	263/1165	0.53(0.43–0.65)
41–45	111/653	1.34(0.97–1.84)	61/603	0.20(0.14–0.27)
P for trend		0.113		< 0.001
*Age at menarche, years*				
≤ 12	205/1531	Reference	532/1858	Reference
13	249/1848	1.00(0.82–1.22)	631/2230	1.04(0.91–1.19)
14	181/1538	0.85(0.68–1.05)	556/1913	1.12(0.97–1.30)
≥ 15	251/1763	1.04(0.85–1.28)	644/2156	1.21(1.05–1.39)
P for trend		> 0.999		0.004
*Parity*				
Nulliparous	617/4559	Reference	1816/5758	Reference
1	100/775	0.91(0.71–1.17)	222/897	0.97(0.82–1.16)
2+	168/1342	0.85(0.67–1.08)	323/1497	1.02(0.85–1.21)
P for trend		0.167		0.901
*Body mass index, kg/m*^2^				
< 18.5	150/1039	1.12(0.92–1.36)	428/1317	1.10(0.97–1.26)
18.5– < 23	586/4464	Reference	1513/5391	Reference
23– < 25	97/684	1.08(0.86–1.37)	232/819	1.14(0.96–1.34)
≥ 25	46/458	0.73(0.53–1.01)	181/593	1.31(1.08–1.58)
P for trend		0.088		0.059
*Vigorous physical activity, METs-hours/week*				
None	378/2727	Reference	966/3315	Reference
Tertile1 (< 1.95)	170/1352	0.89(0.73–1.08)	454/1636	0.87(0.76–1.00)
Tertile2 (1.95–8.90)	161/1267	0.90(0.74–1.10)	507/1613	1.03(0.90–1.17)
Tertile3 (> 8.90)	177/1334	0.94(0.77–1.14)	436/1593	0.83(0.73–0.96)
P for trend		0.718		0.034
Cigarette smoking				
Never	857/6499	Reference	2292/7934	Reference
Ever	29/180	1.23(0.82–1.85)	70/221	1.10(0.83–1.48)
*Alcohol drinking, g/day*				
None	271/2201	Reference	798/2728	Reference
< 5	339/2511	1.12(0.94–1.33)	849/3021	0.89(0.80–1.00)
5– < 15	124/903	1.15(0.91–1.45)	345/1124	0.93(0.80–1.09)
≥ 15	117/763	1.31(1.03–1.66)	266/912	0.84(0.71–0.99)
P for trend		0.053		0.116
*Coffee consumption, cups/day*				
None	90/651	Reference	287/848	Reference
≤ 0.5	383/2804	0.95(0.74–1.21)	1018/3439	0.87(0.73–1.02)
0.5 < –1	183/1504	0.81(0.62–1.07)	552/1873	0.91(0.76–1.09)
> 1	195/1418	0.92(0.70–1.22)	401/1624	0.77(0.64–0.93)
P for trend		0.528		0.030
*Rotating night shifts, nights/month*				
None	288/2076	Reference	597/2385	Reference
< 7	309/2396	0.90(0.75–1.07)	910/2997	1.10(0.97–1.25)
≥ 7	287/2199	0.91(0.75–1.10)	854/2766	1.01(0.88–1.15)
P for trend		0.257		0.637
*On feet at work, hours/day*				
≤ 4	281/2186	Reference	719/2624	Reference
5–8	381/3052	0.98(0.83–1.16)	1080/3751	0.94(0.84–1.05)
≥ 9	224/1442	1.27(1.04–1.56)	564/1782	0.95(0.82–1.09)
P for trend		0.081		0.328
*Heavy lifting at work, times/day*				
0	153/1187	Reference	356/1390	Reference
1–5	509/3899	1.01(0.83–1.23)	1426/4816	1.09(0.95–1.26)
≥ 6	224/1594	1.10(0.88–1.38)	581/1951	1.06(0.90–1.24)
P for trend		0.279		0.954

ORs, odds ratios; CIs, confidence intervals; METs, metabolic equivalents

^a^Compared to women reporting moderate cycle lengths (26–31 days)

Adjusted for age (years), age at menarche (≤ 12, 13, 14, or ≥ 15 years), parity (nulliparous, 1, or 2+), body mass index (kg/m^2^), vigorous physical activity (none or tertiles), and alcohol consumption (0, < 5, 5– < 15, or ≥ 15 g/day)

For sensitivity analyses, we excluded women aged > 40 years or restricted analysis to nulliparous women; the results were similar to those of the main analysis (Additional file 1: Table S2). In nulliparous women, those with a higher frequency of rotating night shifts were more likely to have irregular menstrual cycles (> 7 night shifts per month vs. none: OR 1.19; 95% CI 1.01–1.39, p for trend = 0.032). The results were robust when we restricted analysis to women with a time interval of < 2 years between modules 1 and 3 (data not shown).

Women who perceived their health to be poor were more likely to have irregular menstrual cycles (“poor or very poor” vs. “excellent or good”: OR 1.65; 95% CI 1.41–1.92). Multivariable-adjusted LSmeans of menstrual distress and perceived health status according to the menstrual cycle characteristics are presented in Table 3. The levels of menstrual and premenstrual distress were significantly higher in women with irregular menstrual cycles than in those with regular cycles (*p* < 0.001 for each). The levels of depressive symptoms, perceived stress, physical or mental fatigue, anxiety, and sleep problems were significantly higher in women with irregular cycles than in those with regular cycles (*p* < 0.001 for each). The adjusted LSmeans across the four levels of cycle regularity were significantly increased with increasing variability of cycles (Additional file 1: Table S3). Further, the levels of menstrual and premenstrual distress were significantly lower in women with moderate cycle lengths than in those with short or long cycle lengths (*p* < 0.05 for each, post Bonferroni correction). The levels of perceived stress and mental fatigue were significantly higher in women with short cycle lengths than in those with moderate or long cycle lengths. The results of sensitivity analyses were similar to those of the main analysis (Additional file 1: Table S4).

**Table 3:** Least-square means of menstrual distress and perceived health status according to the menstrual cycle characteristics

Multivariable-adjusted LSmeans ± SE	Menstrual cycle regularity			Menstrual cycle length				
	Regular (n = 7359)	Irregular (n = 1976)	*p* value	< 26 days (n = 886)	26–31 days (n = 5794)	32–50 days (n = 2363)	*p* value	P for trend
*Menstrual distress*								
Most recent flow	31.94 ± 0.55	35.75 ± 0.75	< 0.001	35.78 ± 1.01^a^	31.80 ± 0.58^a,b^	33.54 ± 0.72^b^	< 0.001	0.840
Four days before flow	24.69 ± 0.54	29.47 ± 0.74	< 0.001	29.05 ± 1.00^a^	24.53 ± 0.57^a,b^	27.16 ± 0.70^b^	< 0.001	0.234
*Perceived health status*								
Depressive symptoms	7.01 ± 0.11	7.73 ± 0.15	< 0.001	7.34 ± 0.20	7.04 ± 0.12	7.16 ± 0.14	0.260	0.996
Perceived stress	6.49 ± 0.05	6.82 ± 0.06	< 0.001	6.84 ± 0.09^a,c^	6.52 ± 0.05^a^	6.53 ± 0.06^c^	0.001	0.057
Physical fatigue	12.00 ± 0.08	12.54 ± 0.11	< 0.001	12.17 ± 0.15	12.07 ± 0.09	12.11 ± 0.11	0.783	0.968
Mental fatigue	5.55 ± 0.05	5.84 ± 0.07	< 0.001	5.82 ± 0.10^a^	5.58 ± 0.06^a^	5.60 ± 0.07	0.040	0.255
Anxiety	15.03 ± 0.06	15.46 ± 0.08	< 0.001	15.30 ± 0.11	15.08 ± 0.06	15.09 ± 0.08	0.098	0.292
Sleep problems	10.41 ± 0.10	11.20 ± 0.14	< 0.001	10.39 ± 0.19	10.50 ± 0.11	10.69 ± 0.13	0.202	0.074

Adjusted for age (years), age at menarche (≤ 12, 13, 14, or ≥ 15 years), parity (nulliparous, 1, or 2+), body mass index (kg/m^2^), vigorous physical activity (none or tertiles), and alcohol consumption (0, < 5, 5– < 15, or ≥ 15 g/day)

*Missing data*: menstrual distress-most recent flow (*n* = 1), depressive symptoms (*n* = 9), physical or mental fatigue, anxiety, and sleep problems (*n* = 1 for each)

LSmeans, Least-square means; SE, standard error

^a^Significantly different between < 26 days and 26–31 days (*p* < 0.05, post Bonferroni correction)

^b^Significantly different between 32–50 days and 26–31 days

^c^Significantly different between < 26 days and 32–50 days

## Discussion

In this cross-sectional study, 21% of the women reported irregular menstrual cycles, while 10% and 26% of the women reported short and long menstrual cycle lengths, respectively. Irregular menstrual cycles were associated with young age, late age at menarche, nulliparous, high BMI, and low physical activity; also, with long hours of standing work and frequent heavy lifting. Similar associations were observed in the analyses of short or long cycle length. The levels of menstrual and premenstrual distress were higher in women with irregular and short or long cycle lengths than in women with regular and moderate cycle length. The scores of depressive symptoms, perceived stress, physical or mental fatigue, anxiety, and sleep problems were higher in women with irregular cycles than in those with regular cycles.

To our knowledge, this is the largest study that described the regularity and length of menstrual cycles in young Korean women. The proportion of women reporting irregular cycles in our study was higher than that in the fifth Korea National Health and Nutrition Examination Survey (KNHANES) conducted between 2010 and 2012 (14%) [16, 39]. The differences in questionnaires, occupational status, and time of the survey may contribute to differences in prevalence. In the KNHANES, irregular menstrual cycle was defined as menstruation without any periodic cycle; and shift workers who accounted for < 15% of the participants were 1.39 times more likely to have irregular cycles than daytime workers [39]. Nurses are the largest healthcare workforce [40] and subjected to a challenging work environment (e.g., shift work and physically demanding tasks) [41]. Therefore, the relatively higher prevalence of irregular menstrual cycles in our study population may be largely attributed to their occupational characteristics. Indeed, in the NHS3, the prevalence of irregular menstrual cycles was 19% of 6309 nurses aged 21–45 years between 2010 and 2012 [15], which is consistent with that identified in the present study. Our study results suggest the need for organizational support to provide favorable nursing work environments and assistance to improve reproductive health. According to reports from the National Health Insurance Service–National Health Screening Cohort in Korea, the age-standardized prevalence rate of menstrual disorders in Korean women significantly increased from 8.6% in 2009 to 11.6% in 2016 [42]. Identification of abnormal menstrual patterns may permit early detection of potential health problems [2]. Therefore, the menstrual cycle should be considered as an indicator of health status, and education is required on the normal menstrual cycles and associated modifiable risk factors across the reproductive lifespan.

The length of menstrual cycle generally ranges from 25 to 30 (median 28) days in healthy women and it decreases with age until menopause as the length of follicular phase shortens; and menstrual cycles are often irregular immediately after menarche and shortly before menopause [1, 3, 43]. The distribution of menstrual cycle length in our study was similar to that in a recent study with the largest database of menstrual cycles collected using an app; of the 612,613 menstrual cycles from more than 120,000 users registered between 2016 and 2019, 9%, 65%, and 26% had < 25 days, 25–30 days, and 31–50 days of cycle lengths, respectively [44]. Consistent with previous findings, we observed that the variability and length of the menstrual cycle decreased with increasing age. The results are also consistent with those of other studies that late age at menarche is associated with long or irregular menstrual cycles [11, 13, 33] and that parity is inversely associated with variability of the menstrual cycle [13, 45].

The prevalence of overweight and obesity are increasing globally in all ages and both sexes [46]; thus, the impact of obesity on women’s health across the reproductive lifespan requires more attention. The cutoff point defining obesity differs by region and ethnicity, but epidemiological studies have identified that obese women were more likely to experience long and/or irregular menstrual cycles than non-obese women [11, 13, 33, 36, 47]. Menstrual dysfunction in obese women may be related to insulin resistance/hyperinsulinemia, decreased sex hormone-binding globulin, and alterations in the hypothalamic–pituitary–ovarian axis [47, 48]. In the present study, women with BMI ≥ 25 kg/m^2^ had higher odds of having long and irregular menstrual cycles than those with BMI 18.5– < 23 kg/m^2^, even in the absence of PCOS and other gynecological conditions. Being underweight is also associated with menstrual disorders, and J-shaped associations of body composition measures with long or irregular cycles have been reported in previous studies [47, 49]. In the present study, approximate J-shaped associations of BMI with long and irregular cycles were observed in age-adjusted models, but attenuated after further adjustment for potential confounders.

Weight-loss, athletic activity, and stress have been associated with functional hypothalamic amenorrhea which is characterized by the suppression of gonadotropin-releasing hormone pulsatility [50]. However, recreational physical activity has shown inconsistent associations with regularity and length of menstrual cycles [31, 33, 35, 36]. In our study, recreational physical activity of vigorous intensity was inversely associated with long and irregular menstrual cycles, whereas long hours of standing and frequent heavy lifting at work were associated with irregular cycles. Moreover, prolonged standing work was also associated with short cycles. These findings suggest that both lifestyle modification and improvement in work conditions should be emphasized to improve menstrual health. Indeed, physically demanding work may be associated with menstrual function [15, 51, 52]. Consistent with our study, frequent heavy lifting was significantly associated with irregular cycles in the NHS3 [15]. In that the NHS3 study, prolonged standing and frequent heavy lifting at work tended to be associated with short menstrual cycles.

Associations between shift works and menstrual cycles have been examined based on the hypothesis that disrupted circadian rhythms may play a role in menstrual dysfunction. A recent meta-analysis suggested that women who worked rotating shifts were more likely to experience irregular cycles than those working fixed day shifts and this association was more pronounced among studies with the mean age of < 30 years [17]. The dose–response associations of the number of months worked rotating night shifts over the past 2 years with irregular and long menstrual cycles were observed in the NHS II [13]. In the NHS3, nurses who worked nights only or rotating with nights had a higher prevalence of irregular cycles than those who worked only during the day; also, rotating night shift work was associated with long menstrual cycles [15]. In the present study, more frequent rotating night shifts was associated with irregular cycles in nulliparous women but not in all. Although we adjusted for parity, there may be residual confounding by other factors related to reproductive history. Therefore, further research on the interrelationship between rotating night shifts, menstrual cycle characteristics, and fertility or pregnancy outcome is warranted.

Some studies suggest that cigarette smoking may be associated with short or irregular menstrual cycles mainly by disrupting endocrine function [3, 53]. We found no significant association between lifetime cigarette smoking and menstrual cycle characteristics, but a limited number of smokers may explain the lack of significance. The proportion of never smokers in our study (97%) was much higher than that in the fifth KNHANES (87%) [36]. Mixed results have been reported regarding alcohol consumption and menstrual cycle characteristics [13, 32, 35, 36, 54, 55]. Some previous studies showed that higher alcohol consumption was associated with shorter cycles and/or reduced odds of having long cycles [32, 54], consistent with results of the present study. Women who consumed more coffee were less likely to have long menstrual cycles in the present study, but there are no consistent associations between caffeine intake and menstrual cycles [35, 54, 56]. Thus, further investigation on the role of modifiable lifestyle factors in menstrual function is needed.

Perceived stress [33, 34, 37, 57], depression [11, 37, 57, 58], sleep problem [18, 37, 57], and other psychological symptoms [57, 58] have been associated with irregular menstrual cycles, consistent with our results. We observed that premenstrual and menstrual distress, including physical, emotional, and behavioral symptoms, were higher in women with irregular, short, or long menstrual cycles. Because menstrual distress is correlated with psychological stress, depressive symptoms, and sleep disturbance, further research with a multidimensional approach would help achieve causal inferences and manage reproductive health.

Our study has several limitations. First, the data on menstrual cycle characteristics were self-reported. Low to moderate agreement between reported and observed menstrual cycle lengths have been suggested in other validation studies [59, 60]. However, we collected data on both the regularity and length of cycles as a measure of menstrual patterns, and obtained findings consistent with those of previous studies in terms of age, reproductive and lifestyle factors, and perceived health status. Additionally, the distribution of cycle lengths in our study was similar to that observed in other populations of women in a large-scale, app-based study. Moreover, misclassification of menstrual cycle length or regularity may be non-differential with respect to various exposures. Second, information on some variables, including body weight, work condition, and perceived health status, was collected from the baseline module 1 survey, with a median follow-up of two years. Associations may be attenuated due to the lack of updated information but results were robust when we restricted analyses to women with less than two years of follow up. Nevertheless, future confirmation with updated information from subsequent modules is needed. Third, generalizability to the entire reproductive-age population may be limited because our population consists of female nurses aged between 20 and 45 years. Finally, due to the cross-sectional nature of the study, it was not possible to establish causal relationships. Despite these limitations, this is the largest study to identify menstrual cycle patterns characterized by regularity and length in Korean women. The availability of several factors that have been associated with menstrual cycle characteristics is another strength of our study. The modifiable risk factors identified in this study could be targeted for intervention programs to improve menstrual health.

## Conclusion

Our study provides evidence on menstrual cycle patterns and associated factors in female nurses in Korea. Irregular and long menstrual cycles were associated with young age, late age at menarche, high BMI, and low physical activity. Work conditions, including prolonged standing and frequent heavy lifting, were associated with short and/or irregular menstrual cycles. Women with irregular cycles had higher levels of menstrual distress, depressive symptoms, perceived stress, physical or mental fatigue, anxiety, and sleep problems than those with regular cycles. The findings are generally consistent with the existing literature and may contribute to a better understanding of modifiable risk factors for menstrual dysfunction. The results of this study may serve as a basis for further research on reproductive health.

## Supplementary Information

**Additional file 1**. Supplementary data. Tables S1 to S4.

## Data Availability

The datasets used and analyzed during the current study are available from the corresponding author on reasonable request.

## Abbreviations

KNHS: Korea Nurses’ Health Study
PCOS: Polycystic ovary syndrome
NHS: Nurses’ Health Study
MDQ-C: Menstrual Distress Questionnaire Form-C
MET: Metabolic equivalent
BMI: Body mass index
PSS-4: Four-item Perceived Stress Scale
FFQ: Food frequency questionnaire
OR: Odds ratio
CI: Confidence interval
LSmeans: Least-square means
SE: Standard error
KNHANES: Korea National Health and Nutrition Examination Survey

## References

[1] Reed BG, Carr BR. The normal menstrual cycle and the control of ovulation. 2015.

[2] American Academy of Pediatrics and American College of Obstetricians Gynecologists. Menstruation in girls and adolescents: using the menstrual cycle as a vital sign. In: Pediatrics, vol. 118; 2006. p. 2245–2250. 10.1542/peds.2006-2481 17079600

[3] Harlow SD, Windham G, Paramsothy P. Menstruation and menstrual disorders: the epidemiology of menstruation and menstrual dysfunction. In: Women health, 2nd edition. 2013.

[4] Rostami Dovom M, Ramezani Tehrani F, Djalalinia S, Cheraghi L, Behboudi Gandavani S, Azizi F (2016). Menstrual cycle irregularity and metabolic disorders: a population-based prospective study. PLoS ONE.

[5] Wang YX, Shan Z, Arvizu M, Pan A, Manson JE, Missmer SA, Sun Q, Chavarro JE (2020). Associations of menstrual cycle characteristics across the reproductive life span and lifestyle factors with risk of type 2 diabetes. JAMA Netw Open.

[6] Solomon CG, Hu FB, Dunaif A, Rich-Edwards JE, Stampfer MJ, Willett WC, Speizer FE, Manson JE (2002). Menstrual cycle irregularity and risk for future cardiovascular disease. J Clin Endocrinol Metab.

[7] Gast G-CM, Grobbee DE, Smit HA, Bueno-de-Mesquita HB, Samsioe GN, van der Schouw YT (2010). Menstrual cycle characteristics and risk of coronary heart disease and type 2 diabetes. Fertil Steril.

[8] Cirillo PM, Wang ET, Cedars MI (2016). Chen L-m, Cohn BA: Irregular menses predicts ovarian cancer: prospective evidence from the Child Health and Development Studies. Int J Cancer.

[9] Wang YX, Arvizu M, Rich-Edwards JW, Stuart JJ, Manson JE, Missmer SA, Pan A, Chavarro JE (2020). Menstrual cycle regularity and length across the reproductive lifespan and risk of premature mortality: prospective cohort study. BMJ.

[10] Kato I, Toniolo P, Koenig KL, Shore RE, Zeleniuch-Jacquotte A, Akhmedkhanov A, Riboli E (1999). Epidemiologic correlates with menstrual cycle length in middle aged women. Eur J Epidemiol.

[11] Rowland AS, Baird DD, Long S, Wegienka G, Harlow SD, Alavanja M, Sandler DP (2002). Influence of medical conditions and lifestyle factors on the menstrual cycle. Epidemiology.

[12] Harlow SD, Campbell OM (2004). Epidemiology of menstrual disorders in developing countries: a systematic review. BJOG.

[13] Lawson CC, Whelan EA, Lividoti Hibert EN, Spiegelman D, Schernhammer ES, Rich-Edwards JW (2011). Rotating shift work and menstrual cycle characteristics. Epidemiology.

[14] Lyngsø J, Ramlau-Hansen C, Høyer BB, Støvring H, Bonde JP, Jönsson B, Lindh C, Pedersen H, Ludwicki J, Zviezdai V (2014). Menstrual cycle characteristics in fertile women from Greenland, Poland and Ukraine exposed to perfluorinated chemicals: a cross-sectional study. Hum Reprod.

[15] Lawson CC, Johnson CY, Chavarro JE, Lividoti Hibert EN, Whelan EA, Rocheleau CM, Grajewski B, Schernhammer ES, Rich-Edwards JW (2015). Work schedule and physically demanding work in relation to menstrual function: the Nurses’ Health Study 3. Scand J Work Environ Health.

[16] Jung E-K, Kim S-W, Ock S-M, Jung K-I, Song C-H (2018). Prevalence and related factors of irregular menstrual cycles in Korean women: the 5th Korean National Health and Nutrition Examination Survey (KNHANES-V, 2010–2012). J Psychosom Obstet Gynaecol.

[17] Chang WP, Chang YP (2021). Meta-analysis comparing menstrual regularity and dysmenorrhea of women working rotating shifts and fixed day shifts. J Womens Health (Larchmt).

[18] Kang W, Jang KH, Lim HM, Ahn JS, Park WJ (2019). The menstrual cycle associated with insomnia in newly employed nurses performing shift work: a 12-month follow-up study. Int Arch Occup Environ Health.

[19] Kim JH, Kim O, Jung H, Pang Y, Dan H. Body mass index, menstruation, acne, and hirsutism of polycystic ovary syndrome in women: a cross-sectional study. Health Care Women Int. 2021;1–13. 10.1080/07399332.2021.1939348 34292855

[20] Kim O, Ahn Y, Lee HY, Jang HJ, Kim S, Lee JE, Jung H, Cho E, Lim JY, Kim MJ (2017). The Korea Nurses' Health Study: a prospective cohort study. J Womens Health (Larchmt).

[21] Moos RH (1968). The development of a menstrual distress questionnaire. Psychosom Med.

[22] Moos RH. Menstrual distress questionnaire manual, instrument and scoring guide. Mind Garden, CA, USA. 2010.

[23] Hu FB, Stampfer MJ, Colditz GA, Ascherio A, Rexrode KM, Willett WC, Manson JE (2000). Physical activity and risk of stroke in women. JAMA.

[24] Ainsworth BE, Haskell WL, Whitt MC, Irwin ML, Swartz AM, Strath SJ, O'Brien WL, Bassett DR, Schmitz KH, Emplaincourt PO (2000). Compendium of physical activities: an update of activity codes and MET intensities. Med Sci Sports Exerc.

[25] World Health Organization. The Asia-Pacific perspective: redefining obesity and its treatment. 2000.

[26] Cohen S, Kamarck T, Mermelstein R. A global measure of perceived stress. J Health Soc Behav. 1983;385–396. 6668417

[27] Kroenke K, Spitzer RL, Williams JB (2001). The PHQ-9: validity of a brief depression severity measure. J Gen Intern Med.

[28] Chalder T, Berelowitz G, Pawlikowska T, Watts L, Wessely S, Wright D, Wallace E (1993). Development of a fatigue scale. J Psychosom Res.

[29] Marteau TM, Bekker H (1992). The development of a six-item short-form of the state scale of the Spielberger State—Trait Anxiety Inventory (STAI). Br J Clin Psychol.

[30] Jenkins CD, Stanton B-A, Niemcryk SJ, Rose RM (1988). A scale for the estimation of sleep problems in clinical research. J Clin Epidemiol.

[31] Harlow SD, Matanoski GM (1991). The association between weight, physical activity, and stress and variation in the length of the menstrual cycle. Am J Epidemiol.

[32] Liu Y, Gold EB, Lasley BL, Johnson WO (2004). Factors affecting menstrual cycle characteristics. Am J Epidemiol.

[33] Chang PJ, Chen PC, Hsieh CJ, Chiu LT (2009). Risk factors on the menstrual cycle of healthy Taiwanese college nursing students. Aust N Z J Obstet Gynaecol.

[34] Yamamoto K, Okazaki A, Sakamoto Y, Funatsu M (2009). The relationship between premenstrual symptoms, menstrual pain, irregular menstrual cycles, and psychosocial stress among Japanese college students. J Physiol Anthropol.

[35] Hahn KA, Wise LA, Riis AH, Mikkelsen EM, Rothman KJ, Banholzer K, Hatch EE (2013). Correlates of menstrual cycle characteristics among nulliparous Danish women. Clin Epidemiol.

[36] Bae J, Park S, Kwon JW (2018). Factors associated with menstrual cycle irregularity and menopause. BMC Womens Health.

[37] Kim T, Nam GE, Han B, Cho SJ, Kim J, Eum DH, Lee SW, Min SH, Lee W, Han K (2018). Associations of mental health and sleep duration with menstrual cycle irregularity: a population-based study. Arch Womens Ment Health.

[38] Greenland S (1989). Modeling and variable selection in epidemiologic analysis. Am J Public Health.

[39] Kwak Y, Kim Y (2018). Irregular menstruation according to occupational status. Women Health.

[40] World Health Organization: World health statistics 2016: monitoring health for the SDGs sustainable development goals. World Health Organization; 2016.

[41] Chappel SE, Verswijveren SJJM, Aisbett B, Considine J, Ridgers ND (2017). Nurses’ occupational physical activity levels: a systematic review. Int J Nurs Stud.

[42] Park S, Yoon TW, Kang DR, Chung C (2020). Prevalence of menstrual disorders according to Body Mass Index and lifestyle factors: The National Health Insurance Service-National Health Screening Cohort in Korea, 2009–2016. J Korean Acad Nurs.

[43] Treloar AE (1967). Variation of the human menstrual cycle through reproductive life. Int J Fertil.

[44] Bull JR, Rowland SP, Scherwitzl EB, Scherwitzl R, Danielsson KG, Harper J (2019). Real-world menstrual cycle characteristics of more than 600,000 menstrual cycles. npj Digit Med.

[45] Small CM, Manatunga AK, Klein M, Dominguez CE, Feigelson HS, McChesney R, Marcus M (2010). Menstrual cycle variability and the likelihood of achieving pregnancy. Rev Environ Health.

[46] Chooi YC, Ding C, Magkos F (2019). The epidemiology of obesity. Metabolism.

[47] Wei S, Schmidt MD, Dwyer T, Norman RJ, Venn AJ (2009). Obesity and menstrual irregularity: associations with SHBG, testosterone, and insulin. Obesity (Silver Spring).

[48] Klenov VE, Jungheim ES (2014). Obesity and reproductive function: a review of the evidence. Curr Opin Obstet Gynecol.

[49] Symons JP, Sowers MF, Harlow SD (1997). Relationship of body composition measures and menstrual cycle length. Ann Hum Biol.

[50] Gordon CM (2010). Functional hypothalamic amenorrhea. N Engl J Med.

[51] Messing K, Saurel-Cubizolles M-J, Bourgine M, Kaminski M. Menstrual-cycle characteristics and work conditions of workers in poultry slaughterhouses and canneries. Scand J Work Environ Health. 1992;302–309. 10.5271/sjweh.1572 1439657

[52] Jeyaseelan L, Rao PSS (1995). Effect of occupation on menstrual cycle length: causal model. Hum Biol.

[53] Windham GC, Mitchell P, Anderson M, Lasley BL (2005). Cigarette smoking and effects on hormone function in premenopausal women. Environ Health Perspect.

[54] Cooper GS, Sandler DP, Whelan EA, Smith KR. Association of physical and behavioral characteristics with menstrual cycle patterns in women age 29–31 years. Epidemiology. 1996;624–628. 10.1097/00001648-199611000-00010 8899389

[55] Lyngsø J, Toft G, Høyer B, Guldbrandsen K, Olsen J, Ramlau-Hansen C (2014). Moderate alcohol intake and menstrual cycle characteristics. Hum Reprod.

[56] Fenster L, Quale C, Waller K, Windham GC, Elkin EP, Benowitz N, Swan SH (1999). Caffeine consumption and menstrual function. Am J Epidemiol.

[57] Kennedy KER, Onyeonwu C, Nowakowski S, Hale L, Branas CC, Killgore WDS, Wills CCA, Grandner MA (2021). Menstrual regularity and bleeding is associated with sleep duration, sleep quality and fatigue in a community sample. J Sleep Res.

[58] Toffol E, Koponen P, Luoto R, Partonen T (2014). Pubertal timing, menstrual irregularity, and mental health: results of a population-based study. Arch Womens Ment Health.

[59] Small CM, Manatunga AK, Marcus M (2007). Validity of self-reported menstrual cycle length. Ann Epidemiol.

[60] Jukic AM, Weinberg CR, Wilcox AJ, McConnaughey DR, Hornsby P, Baird DD (2008). Accuracy of reporting of menstrual cycle length. Am J Epidemiol.

