# Supplementary material for: Factors associated with regularity and length of menstrual cycle: Korea Nurses’ Health Study
Source: BMC Womens Health. 2022 Sep 1;22:361. doi: 10.1186/s12905-022-01947-z (PMC9438137; doi:10.1186/s12905-022-01947-z)
Supplement: Supplementary file 1 — Additional file 1. Supplementary data. Tables S1 to S4. [file 12905_2022_1947_MOESM1_ESM.docx]

Additional file 1

**Supplementary Table 1.** Age-and multivariable-adjusted associations of reproductive, lifestyle, and occupational factors with menstrual cycle irregularity

|  | No of irregular cycles/ total | Irregular menstrual cycles ^a^ | |
| --- | --- | --- | --- |
| ORs (95% CIs) |  | Age-adjusted | Multivariable-adjusted ^b^ |
| Age, years |  |  |  |
| 22–25 | 498/1679 | Reference | Reference |
| 26–30 | 849/3602 | 0.73(0.64-0.83) | 0.74(0.65-0.84) |
| 31–35 | 373/2020 | 0.54(0.46-0.63) | 0.59(0.50-0.70) |
| 36–40 | 162/1303 | 0.34(0.28-0.41) | 0.39(0.31-0.49) |
| 41–45 | 94/731 | 0.35(0.28-0.45) | 0.41(0.31-0.54) |
| P for trend |  | <0.001 | <0.001 |
| Age at menarche, years |  |  |  |
| ≤12 | 430/2139 | Reference | Reference |
| 13 | 511/2544 | 1.05(0.91-1.22) | 1.08(0.93-1.25) |
| 14 | 426/2157 | 1.07(0.92-1.24) | 1.11(0.96-1.30) |
| ≥15 | 609/2495 | 1.46(1.27-1.68) | 1.53(1.32-1.77) |
| P for trend |  | <0.001 | <0.001 |
| Parity |  |  |  |
| Nulliparous | 1601/6620 | Reference | Reference |
| 1 | 163/1023 | 0.80(0.66-0.96) | 0.77(0.64-0.93) |
| 2+ | 210/1686 | 0.72(0.59-0.88) | 0.69(0.57-0.84) |
| P for trend |  | <0.001 | <0.001 |
| Body mass index, kg/m^2^ |  |  |  |
| <18.5 | 364/1522 | 1.14(1.00-1.31) | 1.07(0.93-1.23) |
| 18.5–<23 | 1231/6132 | Reference | Reference |
| 23–<25 | 193/944 | 1.14(0.96-1.36) | 1.19(1.00-1.42) |
| ≥25 | 177/689 | 1.60(1.33-1.92) | 1.68(1.40-2.03) |
| P for trend |  | <0.001 | <0.001 |
| Vigorous physical activity,  METs-hours/week |  |  |  |
| None | 816/3813 | Reference | Reference |
| Tertile1 (<1.95) | 401/1858 | 0.93(0.81-1.07) | 0.93(0.81-1.07) |
| Tertile2 (1.95–8.90) | 379/1823 | 0.87(0.76-1.00) | 0.86(0.75-0.99) |
| Tertile3 (>8.90) | 380/1841 | 0.85(0.74-0.98) | 0.83(0.72-0.96) |
| P for trend |  | 0.038 | 0.017 |
| Cigarette smoking |  |  |  |
| Never | 1913/9073 | Reference | Reference |
| Ever | 62/260 | 1.11(0.83-1.49) | 1.08(0.81-1.46) |
| Alcohol drinking, g/day |  |  |  |
| None | 637/3087 | Reference | Reference |
| <5 | 691/3448 | 0.90(0.80-1.02) | 0.90(0.79-1.01) |
| 5–<15 | 294/1295 | 0.96(0.81-1.12) | 0.95(0.81-1.12) |
| ≥15 | 259/1079 | 1.00(0.84-1.18) | 0.98(0.82-1.16) |
| P for trend |  | 0.536 | 0.720 |
| Coffee consumption, cups/day |  |  |  |
| None | 239/977 | Reference | Reference |
| ≤0.5 | 838/3951 | 0.85(0.72-1.00) | 0.87(0.73-1.03) |
| 0.5<–1 | 434/2108 | 0.87(0.73-1.04) | 0.89(0.74-1.07) |
| ≥1 | 370/1872 | 0.91(0.75-1.10) | 0.92(0.75-1.11) |
| P for trend |  | 0.942 | 0.995 |
| Rotating night shifts, nights/month |  |  |  |
| None | 475/2734 | Reference | Reference |
| <7 | 736/3409 | 1.08(0.95-1.23) | 1.06(0.92-1.21) |
| ≥7 | 761/3180 | 1.10(0.96-1.26) | 1.06(0.92-1.22) |
| P for trend |  | 0.149 | 0.373 |
| On feet at work, hours/day |  |  |  |
| ≤4 | 547/2989 | Reference | Reference |
| 5–8 | 886/4254 | 1.02(0.90-1.15) | 1.02(0.91-1.16) |
| ≥9 | 543/2092 | 1.19(1.04-1.38) | 1.20(1.04-1.38) |
| P for trend |  | 0.038 | 0.036 |
| Heavy lifting at work, times/day |  |  |  |
| 0 | 287/1590 | Reference | Reference |
| 1–5 | 1143/5472 | 1.06(0.92-1.23) | 1.05(0.91-1.22) |
| ≥6 | 546/2273 | 1.22(1.04-1.43) | 1.20(1.01-1.41) |
| P for trend |  | 0.007 | 0.014 |

*Abbreviations*: ORs, odds ratios; CIs, confidence intervals; METs, metabolic equivalents.

^a^ Compared to regular cycles defined as ≤7-day variability between cycles.

^b^ Adjusted for age (years), age at menarche (≤12, 13, 14, or ≥15 years), parity (nulliparous, 1, or 2+), body mass index (kg/m^2^), vigorous physical activity (none or tertiles), and alcohol consumption (0, <5, 5–<15, or ≥15g/day).

**Supplementary Table 2.** Sensitivity analyses for the associations of menstrual cycle characteristics with reproductive, lifestyle, and occupational factors

| Multivariable-adjusted  ORs (95% CIs) ^a^ | Women aged 40 years or less (n=8604) | | |  | Nulliparous women (n=6620) | | |
| --- | --- | --- | --- | --- | --- | --- | --- |
|  | Irregular cycle (n=1182) | Short length (n=775) | Long length (n=2302) |  | Irregular cycle (n=1601) | Short length (n=617) | Long length (n=1816) |
| Age, years |  |  |  |  |  |  |  |
| 22–25 | Reference | Reference | Reference |  | Reference | Reference | Reference |
| 26–30 | 0.74(0.65-0.84) | 0.72(0.59-0.88) | 0.97(0.85-1.11) |  | 0.75(0.65-0.85) | 0.73(0.60-0.90) | 0.98(0.86-1.12) |
| 31–35 | 0.59(0.50-0.70) | 0.82(0.65-1.05) | 0.73(0.62-0.86) |  | 0.58(0.48-0.69) | 0.81(0.63-1.05) | 0.72(0.60-0.86) |
| 36–40 | 0.39(0.31-0.49) | 0.86(0.64-1.16) | 0.53(0.43-0.66) |  | 0.30(0.21-0.42) | 0.80(0.55-1.18) | 0.44(0.33-0.60) |
| 41–45 | - | - | - |  | 0.38(0.23-0.62) | 1.19(0.71-1.98) | 0.17(0.08-0.34) |
| P for trend | <0.001 | 0.440 | <0.001 |  | <0.001 | 0.669 | <0.001 |
| Menarche age, years |  |  |  |  |  |  |  |
| ≤12 | Reference | Reference | Reference |  | Reference | Reference | Reference |
| 13 | 1.08(0.93-1.25) | 0.97(0.78-1.19) | 1.04(0.91-1.20) |  | 1.10(0.93-1.29) | 0.99(0.79-1.25) | 1.01(0.86-1.18) |
| 14 | 1.12(0.96-1.31) | 0.85(0.68-1.07) | 1.15(0.99-1.33) |  | 1.10(0.92-1.30) | 0.87(0.68-1.13) | 1.16(0.99-1.37) |
| ≥15 | 1.52(1.31-1.76) | 1.03(0.83-1.28) | 1.22(1.05-1.40) |  | 1.49(1.26-1.75) | 0.98(0.77-1.25) | 1.18(1.00-1.38) |
| P for trend | <0.001 | 0.975 | 0.003 |  | <0.001 | 0.643 | 0.015 |
| Parity |  |  |  |  |  |  |  |
| Nulliparous | Reference | Reference | Reference |  | - | - | - |
| 1 | 0.78(0.63-0.95) | 0.96(0.73-1.26) | 0.92(0.77-1.11) |  | - | - | - |
| 2+ | 0.71(0.57-0.88) | 0.86(0.65-1.14) | 1.00(0.84-1.21) |  | - | - | - |
| P for trend | 0.001 | 0.319 | 0.903 |  | - | - | - |
| Body mass index, kg/m^2^ |  |  |  |  |  |  |  |
| <18.5 | 1.07(0.93-1.23) | 1.07(0.87-1.31) | 1.09(0.95-1.24) |  | 1.03(0.89-1.19) | 1.15(0.92-1.43) | 1.10(0.96-1.28) |
| 18.5–<23 | Reference | Reference | Reference |  | Reference | Reference | Reference |
| 23–<25 | 1.24(1.03-1.49) | 1.09(0.84-1.41) | 1.08(0.91-1.28) |  | 1.23(1.00-1.51) | 1.23(0.92-1.65) | 1.02(0.83-1.26) |
| ≥25 | 1.66(1.36-2.03) | 0.77(0.54-1.10) | 1.30(1.07-1.59) |  | 1.73(1.38-2.17) | 0.72(0.47-1.11) | 1.21(0.95-1.53) |
| P for trend | <0.001 | 0.273 | 0.114 |  | <0.001 | 0.258 | 0.729 |
| Vigorous physical activity, METs-hours/week |  |  |  |  |  |  |  |
| None | Reference | Reference | Reference |  | Reference | Reference | Reference |
| Tertile1 (<1.95) | 0.95(0.82-1.09) | 0.85(0.69-1.04) | 0.88(0.77-1.01) |  | 0.94(0.81-1.10) | 0.92(0.72-1.16) | 0.90(0.77-1.06) |
| Tertile2 (1.95–8.90) | 0.86(0.74-0.99) | 0.89(0.72-1.10) | 1.05(0.92-1.20) |  | 0.86(0.74-1.01) | 0.84(0.66-1.06) | 1.06(0.91-1.24) |
| Tertile3 (>8.90) | 0.84(0.73-0.97) | 0.93(0.75-1.14) | 0.87(0.75-1.00) |  | 0.84(0.72-0.98) | 0.92(0.74-1.16) | 0.87(0.75-1.01) |
| P for trend | 0.025 | 0.798 | 0.126 |  | 0.037 | 0.649 | 0.140 |
| Cigarette smoking |  |  |  |  |  |  |  |
| Never | Reference | Reference | Reference |  | Reference | Reference | Reference |
| Ever | 1.11(0.82-1.51) | 1.17(0.76-1.80) | 1.09(0.81-1.47) |  | 1.06(0.76-1.48) | 1.12(0.68-1.85) | 1.08(0.77-1.51) |

**Supplementary Table 2.** *Continued*

| Multivariable-adjusted  ORs (95% CIs) ^a^ | Women aged 40 years or less (n=8604) | | |  | Nulliparous women (n=6620) | | |
| --- | --- | --- | --- | --- | --- | --- | --- |
|  | Irregular cycle (n=1182) | Short length (n=775) | Long length (n=2302) |  | Irregular cycle (n=1601) | Short length (n=617) | Long length (n=1816) |
| Alcohol drinking, g/day |  |  |  |  |  |  |  |
| None | Reference | Reference | Reference |  | Reference | Reference | Reference |
| <5 | 0.88(0.78-1.00) | 1.08(0.90-1.31) | 0.89(0.79-1.01) |  | 0.87(0.75-1.00) | 0.96(0.78-1.19) | 0.91(0.79-1.05) |
| 5–<15 | 0.92(0.78-1.09) | 1.06(0.83-1.35) | 0.91(0.78-1.06) |  | 0.92(0.77-1.10) | 0.96(0.73-1.25) | 0.90(0.76-1.07) |
| ≥15 | 0.96(0.81-1.15) | 1.28(1.00-1.64) | 0.83(0.70-0.99) |  | 0.94(0.78-1.13) | 1.11(0.85-1.46) | 0.84(0.70-1.02) |
| P for trend | 0.802 | 0.073 | 0.105 |  | 0.929 | 0.319 | 0.151 |
| Coffee consumption, cups/day |  |  |  |  |  |  |  |
| None | Reference | Reference | Reference |  | Reference | Reference | Reference |
| ≤0.5 | 0.86(0.72-1.02) | 0.95(0.73-1.23) | 0.87(0.74-1.03) |  | 0.86(0.71-1.04) | 0.99(0.74-1.32) | 0.89(0.74-1.08) |
| 0.5<–1 | 0.87(0.72-1.05) | 0.80(0.60-1.07) | 0.90(0.75-1.07) |  | 0.94(0.76-1.16) | 0.88(0.63-1.22) | 0.91(0.74-1.12) |
| >1 | 0.91(0.74-1.11) | 0.91(0.68-1.23) | 0.76(0.63-0.92) |  | 0.92(0.74-1.14) | 0.89(0.63-1.24) | 0.78(0.63-0.97) |
| P for trend | 0.966 | 0.466 | 0.018 |  | 0.781 | 0.293 | 0.044 |
| Rotating night shift, nights/month |  |  |  |  |  |  |  |
| None | Reference | Reference | Reference |  | Reference | Reference | Reference |
| <7 | 1.10(0.96-1.27) | 0.89(0.73-1.08) | 1.08(0.95-1.22) |  | 1.18(1.00-1.39) | 0.87(0.69-1.09) | 1.16(0.99-1.36) |
| >7 | 1.09(0.94-1.25) | 0.88(0.72-1.08) | 0.98(0.86-1.13) |  | 1.19(1.01-1.39) | 0.88(0.71-1.11) | 1.10(0.94-1.28) |
| P for trend | 0.207 | 0.177 | 0.923 |  | 0.032 | 0.253 | 0.197 |
| On feet at work, hours/day |  |  |  |  |  |  |  |
| ≤4 | Reference | Reference | Reference |  | Reference | Reference | Reference |
| 5–8 | 1.03(0.91-1.17) | 0.95(0.79-1.15) | 0.95(0.84-1.07) |  | 1.05(0.91-1.21) | 1.03(0.83-1.28) | 0.89(0.78-1.03) |
| ≥9 | 1.20(1.04-1.39) | 1.24(1.01-1.54) | 0.97(0.85-1.12) |  | 1.26(1.07-1.48) | 1.35(1.06-1.71) | 0.93(0.80-1.09) |
| P for trend | 0.033 | 0.140 | 0.567 |  | 0.009 | 0.032 | 0.274 |
| Heavy lifting at work, times/day |  |  |  |  |  |  |  |
| 0 | Reference | Reference | Reference |  | Reference | Reference | Reference |
| 1–5 | 1.09(0.94-1.28) | 0.99(0.8-1.23) | 1.09(0.95-1.26) |  | 1.04(0.88-1.24) | 1.04(0.80-1.34) | 1.04(0.88-1.23) |
| ≥16 | 1.25(1.05-1.49) | 1.08(0.85-1.38) | 1.06(0.90-1.25) |  | 1.21(1.00-1.46) | 1.19(0.90-1.58) | 1.03(0.85-1.24) |
| P for trend | 0.006 | 0.336 | 0.893 |  | 0.015 | 0.133 | 0.989 |

*Abbreviations*: ORs, odds ratios; CIs, confidence intervals; METs, metabolic equivalents.

^a^ Compared to regular cycles defined as ≤7-days variability between cycles or moderate cycle length (26–31 days).

Adjusted for age (years), age at menarche (≤12, 13, 14, or ≥15 years), parity (nulliparous, 1, or 2+); except for analysis of nulliparous women, body mass index (kg/m^2^), vigorous physical activity (none or tertiles), and alcohol consumption (0, <5, 5–<15, or ≥15g/day).

**Supplementary Table 3.** Least-square means of menstrual distress and perceived health status according to menstrual cycle regularity

| Multivariable-adjusted | Very regular | Regular | Usually irregular | Always irregular |  |
| --- | --- | --- | --- | --- | --- |
| LSmeans±SE | (n=3168) | (n=4191) | (n=1469) | (n=507) | P for trend |
| Menstrual distress |  |  |  |  |  |
| Most recent flow | 29.81±0.65 | 33.59±0.61 | 36.32±0.83 | 34.51±1.27 | <0.001 |
| Four days before flow | 22.70±0.64 | 26.24±0.60 | 30.19±0.82 | 27.76±1.25 | <0.001 |
| Perceived health status |  |  |  |  |  |
| Depressive symptoms | 6.80±0.13 | 7.17±0.12 | 7.66±0.17 | 7.97±0.25 | <0.001 |
| Perceived stress | 6.36±0.06 | 6.59±0.05 | 6.79±0.07 | 6.91±0.11 | <0.001 |
| Physical fatigue | 11.85±0.10 | 12.11±0.09 | 12.55±0.12 | 12.51±0.19 | <0.001 |
| Mental fatigue | 5.46±0.06 | 5.62±0.06 | 5.87±0.08 | 5.75±0.12 | <0.001 |
| Anxiety | 14.87±0.07 | 15.15±0.07 | 15.40±0.09 | 15.65±0.14 | <0.001 |
| Sleep problem | 10.15±0.12 | 10.61±0.12 | 11.08±0.16 | 11.58±0.24 | <0.001 |

*Abbreviations*: LSmeans, Least-square means; SE, standard error.

Adjusted for age (years), age at menarche (≤12, 13, 14, or ≥15 years), parity (nulliparous, 1, or 2+), body mass index (kg/m^2^), vigorous physical activity (none or tertiles), and alcohol consumption (0, <5, 5–<15, or ≥15g/day).

*Missing data*: menstrual distress-most recent flow (*n* = 1), depressive symptoms (*n* = 9), physical or mental fatigue, anxiety, and sleep problems (*n* = 1 for each).

**Supplementary Table 4.** Sensitivity analyses for least-square means of menstrual distress and perceived health status according to menstrual cycle characteristics

| Multivariable-adjusted | Menstrual cycle regularity | |  |  | Menstrual cycle length | | |  |  |
| --- | --- | --- | --- | --- | --- | --- | --- | --- | --- |
| LSmeans±SE | Regular | Irregular | P value |  | <26 days | 26-31 days | 32-50 days | P value | P for trend |
| Women aged 40 years or less (n=8604) |  |  |  |  |  |  |  |  |  |
| Menstrual distress |  |  |  |  |  |  |  |  |  |
| Most recent flow | 32.52±0.60 | 36.07±0.80 | <0.001 |  | 36.35±1.10^a^ | 32.34±0.64^a,b^ | 34.09±0.76^b^ | <0.001 | 0.736 |
| Four days before flow | 25.19±0.60 | 29.71±0.79 | <0.001 |  | 29.53±1.09^a^ | 24.98±0.63^a,b^ | 27.58±0.75^b^ | <0.001 | 0.209 |
| Perceived health status |  |  |  |  |  |  |  |  |  |
| Depressive symptoms | 7.15±0.12 | 7.89±0.16 | <0.001 |  | 7.49±0.22 | 7.20±0.13 | 7.28±0.15 | 0.353 | 0.847 |
| Perceived stress | 6.55±0.05 | 6.89±0.07 | <0.001 |  | 6.95±0.09^a,c^ | 6.59±0.05^a^ | 6.59±0.06^c^ | <0.001 | 0.026 |
| Physical fatigue | 12.10±0.09 | 12.68±0.12 | <0.001 |  | 12.36±0.16 | 12.19±0.09 | 12.21±0.11 | 0.551 | 0.666 |
| Mental fatigue | 5.59±0.06 | 5.89±0.08 | <0.001 |  | 5.89±0.11^a^ | 5.63±0.06^a^ | 5.63±0.07 | 0.030 | 0.155 |
| Anxiety | 15.14±0.07 | 15.57±0.09 | <0.001 |  | 15.44±0.12 | 15.19±0.07 | 15.20±0.08 | 0.091 | 0.280 |
| Sleep problem | 10.52±0.11 | 11.35±0.15 | <0.001 |  | 10.51±0.21 | 10.63±0.12 | 10.79±0.14 | 0.320 | 0.131 |
| Nulliparous women (n=6620) |  |  |  |  |  |  |  |  |  |
| Menstrual distress |  |  |  |  |  |  |  |  |  |
| Most recent flow | 36.33±0.57 | 39.53±0.79 | <0.001 |  | 39.50±1.19^a^ | 36.50±0.62^a^ | 36.99±0.78 | 0.043 | 0.425 |
| Four days before flow | 27.80±0.56 | 32.08±0.78 | <0.001 |  | 31.33±1.17^a^ | 27.93±0.61^a^ | 29.57±0.76 | 0.005 | 0.681 |
| Perceived health status |  |  |  |  |  |  |  |  |  |
| Depressive symptoms | 8.26±0.11 | 8.96±0.16 | <0.001 |  | 8.66±0.24 | 8.33±0.12 | 8.39±0.16 | 0.380 | 0.720 |
| Perceived stress | 6.73±0.05 | 7.05±0.07 | <0.001 |  | 7.12±0.10^a,c^ | 6.77±0.05^a^ | 6.75±0.06^c^ | 0.001 | 0.023 |
| Physical fatigue | 12.38±0.08 | 12.98±0.11 | <0.001 |  | 12.58±0.17 | 12.48±0.09 | 12.46±0.11 | 0.820 | 0.633 |
| Mental fatigue | 5.68±0.05 | 5.97±0.08 | <0.001 |  | 6.01±0.11^a,c^ | 5.72±0.06^a^ | 5.70±0.07^c^ | 0.030 | 0.103 |
| Anxiety | 15.54±0.06 | 16.00±0.09 | <0.001 |  | 15.83±0.13 | 15.63±0.07 | 15.58±0.08 | 0.217 | 0.163 |
| Sleep problem | 11.45±0.11 | 12.32±0.15 | <0.001 |  | 11.50±0.22 | 11.56±0.12 | 11.73±0.15 | 0.462 | 0.218 |

*Abbreviations*: LSmeans, Least-square means; SE, standard error.

Adjusted for age (years), age at menarche (≤12, 13, 14, or ≥15 years), parity (nulliparous, 1, or 2+); except for analysis of nulliparous women, body mass index (kg/m^2^), vigorous physical activity (none or tertiles), and alcohol consumption (0, <5, 5–<15, or ≥15g/day).

^a^ Significantly different between <26 days and 26–31 days (p < 0.05, post Bonferroni correction)

^b^ Significantly different between 32–50 days and 26–31 days

^c^ Significantly different between <26 days and 32–50 days

*Missing data*: menstrual distress-most recent flow (*n* = 1), depressive symptoms (*n* = 9), physical or mental fatigue, anxiety, and sleep problems (*n* = 1 for each).
